# Evaluation of 6 years of eHealth data in the alcohol use disorder field indicates improved efficacy of care

**DOI:** 10.3389/fdgth.2023.1282022

**Published:** 2024-01-05

**Authors:** Mats Wallden, Gunnar Dahlberg, Johan Månflod, Rajna Knez, Maria Winkvist, Andreas Zetterström, Karl Andersson, Markku D. Hämäläinen, Fred Nyberg

**Affiliations:** ^1^ Skillsta Teknik Design och Kvalitet AB, Vänge, Sweden; ^2^ Kontigo Care AB, Uppsala, Sweden; ^3^ Region Uppsala, Needle Exchange Programme, Uppsala, Sweden; ^4^ School of Health Sciences, University of Skövde, Skövde, Sweden; ^5^ Skaraborg Hospital, Skövde, Sweden; ^6^ Department of Immunology, Genetics and Pathology, Uppsala University, Uppsala, Sweden; ^7^ Department of Pharmaceutical Biosciences, Uppsala University, Uppsala, Sweden

**Keywords:** addiction, eHealth, prediction, relapse, alcohol

## Abstract

**Background:**

Predictive eHealth tools will change the field of medicine, however long-term data is scarce. Here, we report findings on data collected over 6 years with an AI-based eHealth system for supporting the treatment of alcohol use disorder.

**Methods:**

Since the deployment of Previct Alcohol, structured data has been archived in a data warehouse, currently comprising 505,641 patient days. The frequencies of relapse and caregiver-patient messaging over time was studied. The effects of both introducing an AI-driven relapse prediction tool and the COVID-19 pandemic were analyzed.

**Results:**

The relapse frequency per patient day among Previct Alcohol users was 0.28 in 2016, 0.22 in 2020 and 0.25 in 2022 with no drastic change during COVID-19. When a relapse was predicted, the actual occurrence of relapse in the days immediately after was found to be above average. Additionally, there was a noticeable increase in caregiver interactions following these predictions. When caregivers were not informed of these predictions, the risk of relapse was found to be higher compared to when the prediction tool was actively being used. The prediction tool decreased the relapse risk by 9% for relapses that were of short duration and by 18% for relapses that lasted more than 3 days.

**Conclusions:**

The eHealth system Previct Alcohol allows for high resolution measurements, enabling precise identifications of relapse patterns and follow up on individual and population-based alcohol use disorder treatment. eHealth relapse prediction aids the caregiver to act timely, which reduces, delays, and shortens relapses.

## 1 Introduction

Recent years have seen an increasing interest for electronic health (eHealth) solutions and digitalized health care systems reflected by a steady growth of available eHealth systems for use in professional health care. Although the use of eHealth interventions in patients with somatic and mental diseases has increased considerably over the past years, reports on effects of services are still limited, in particular for the prevention and treatment of substance use disorders. Nevertheless, internet-based and app-based interventions have to some extent already been directed to alcohol use disorders (AUD). In most cases, these systems are novel so that long-term follow-ups of their utility are rare, leaving the actual effects on patients and care providers unknown. One exception is the app-based eHealth system Previct® Alcohol, providing therapeutical support in AUD (1).

Previct Alcohol is an eHealth system for the support of therapy in confirmed AUD, which has been available as a medical device for use in health care since late 2015. This means that there is more than 6 years of experience and recorded data associated with this system. This device consists of a portable breathalyzer, an application for smartphones (for patients) and a web-based portal (for caregivers). The caregiver plans therapy, controls and monitors the patient's status through the web portal, while patients use the app to perform scheduled activities. Typically, the patient conducts scheduled breath tests, answers questionnaires related to mood, motivation, and wellbeing, reports when craving to consume alcohol occurs, keep a diary, and so on. The data is collected, compiled into digital biomarkers, and presented to the caregiver in a manner that provides insights related to the current status of the patient. The digital biomarkers include both sobriety estimate (1) and a definition of relapse referred to as an exacerbation event (EE) that allows for a more objective analysis of behavioral patterns (2). With this information, care can be individualized by the therapist. In parallel with extensive actual use in the clinic, different other aspects of the Previct Alcohol system have been discussed and reported in several studies (3–5).

According to European Monitoring Centre for Drugs and Drug Addiction, AUD is a condition that affects millions of people only in Europe (6). According to the World Health Organization, around 3 million deaths every year result from harmful use of alcohol. The National Institute of Drug Abuse in the US describes a number of health consequences related to AUD. These include liver damage, heart disease, cancer, as well as social and personal harms such as strained relationships, financial difficulties, mental health problems and criminal behavior (7). The prevalence of hazardous alcohol consumption among 16–84-year-old inhabitants in Sweden is 15 percent. Hazardous consumption is more common among young people than among older people, and among men than among women, as reported by The Public Health Agency of Sweden (2021).

AUD is described as a chronic relapsing brain disease characterized by impaired ability to stop or control alcohol use despite adverse consequences (8, 9). Exposure to stress and alcohol cues contribute significantly to relapse risk. A dysregulation of the hypothalamic-pituitary-adrenal axis followed by increased anxiety and high alcohol craving are often seen during early alcohol recovery and is suggested to influence the risk of relapse (10). Moreover, brain imaging studies indicate that dysfunction of dopaminergic, glutamatergic and opioidergic neurotransmission in the brain reward system (ventral striatum including the nucleus accumbens) can be associated with alcohol craving and functional brain activation in neuronal systems that process attentional relevant stimuli, reward expectancy and experience (11). In addition, recent research reports that relapse occurs in more than fifty percent of newly abstinent patients with alcohol disorder already within 3 months (9). This indicates that prevention of relapse is a main challenge of AUD treatment.

Relapse is considered as a gradual process with distinct stages (12, 13). Therefore, the goal of treatment is to help individuals to recognize the early stages, in which the chances of successful relapse prevention are greatest (14). One factor contributing to relapse is withdrawal-related anxiety, which likely reflects adaptive changes in the brain in response to continued alcohol exposure. These changes affect, for example, the body's stress response system. The relationship between withdrawal, stress, and relapse also has implications for the treatment of individuals with AUD (15). With no further intervention, relapse rates in detoxified alcoholics are high and usually exceed eighty percent of all detoxified patients. It has also been suggested that stress and exposure to priming doses of alcohol and to alcohol-associated stimuli (cues) contribute to the relapse risk after detoxification (11).

Clinical studies have demonstrated that individuals with AUD are more sensitive to relapse-provoking cues and stimuli, such as the portrayal of the drug and drinking behavior, than individuals without AUD (15). The presence of alcohol cues induces physiological reactivity and craving. Additionally, studies have also shown that cue reactivity and craving were positively correlated, and cue reactivity was larger for patients with shorter histories of alcohol dependence (11). Further studies have identified heterogeneity in patterns of remission/relapse to heavy drinking during the first year of after outpatient treatment. These authors reported that patients with continuous remission or transition to remission had better long-term outcome than those with transition to relapse or continuous relapse (16).

As for biomarkers for AUD and its relapse only a few studies have been reported in literature. In addition to the digital biomarkers developed and reported by the Previct R&D team (1, 2), Deng and co-workers recently reported use of a data-driven research to investigate resting-state functional magnetic resonance imaging during early abstinence from alcohol dependence and its potential ability to predict relapse (17). They applied fractional amplitude of low-frequency fluctuation as an imaging biomarker for relapse and found that during a 6 month following-up period around fifty percent of their AUD patients exhibited reduction in some brain regions. The baseline functional connectivity of the left precentral region to the left cerebellum of the relapse group was significantly lower than that of the non-relapse group (17).

It is obvious that once diagnosed with AUD, the challenge of maintaining sobriety or conversely of avoiding relapses emerges and patients will often relapse, having periods of harmful drinking. Hence, the practice of active monitoring of sobriety has been frequently employed use in implemented in many forms. However, successful outcome in therapy varies and improvements are needed. The assumption of our studies on use of the Previct-app is that it is fundamental to immediately notify caretakers or relatives about an oncoming relapse. This enables therapists and indeed the patients themselves to prevent or decrease the magnitude of that relapse.

In May 2021, at the time of the COVID-19 pandemic, a predictor capable of forecasting relapses ahead of time was introduced in Previct Alcohol (denoted the relapse predictor). In brief, data from recent 1–7 weeks is aggregated and used to estimate the relapse risk in the up-coming 1–3 days. The relapse predictor relies on a wide range of input data, and combines breathalyzer results, questionnaire input, patient reported cravings, compliance to tasks, and many other aspects to estimate relapse risk. This allows additional possibilities for individualization and precision care efforts. Worldwide, the COVID-19 pandemic had a considerable impact on alcohol use, with an increase in alcohol related emergencies and changes in alcohol use patterns (18). Also, individuals with AUD were at greater risk for relapse into drinking and had an increased risk of contracting the disease (19). This must be kept in mind when interpreting characteristics over time.

Here, we report an analysis of data collected over the lifetime of Previct Alcohol for the purpose of illustrating the capacities of eHealth solutions in general and Previct Alcohol in particular. The impact of introducing the relapse predictor is analyzed in detail. Thus, the dimensions analyzed were relapse frequency, caregiver-patient messaging frequency and timing of relapse predictions. Their relationship to time, treatment time and to each other were analyzed.

## 2 Materials and methods

When a patient discontinues treatment using the Previct Alcohol device, a subset of the data related to the patient is moved from the production database to a separate data warehouse in an anonymous manner. The data warehouse therefore contains data which is of anonymous register character. Most of the patients use Previct Alcohol 3–12 months, leading to an inherent delay of data fed to the data warehouse. No curation of data was made, this to reflect the cohort in its entirety. During analysis, available data was randomized into multiple partitions. Every statistical claim was first evaluated on about 50%–70% of the data followed by a confirmation on 20%–40% of the data. Consequently, all reported findings have been confirmed on novel data. Extracted data was analyzed using regular descriptive statistics.

It is important to remember that the data collected comes from live use of an eHealth tool, without interventions, randomizations, patient stratification/inclusion/exclusion criteria, treatment arms and similar concepts available in clinical study designs. The data set is a current and plain view of the cohort under active treatment.

As a side note, of the available data (505,641 patient days) a small fraction (∼25%, evenly spread over time, randomly selected) was entirely set aside and not used for the analyses presented in this report. This allows unbiased data analysis in future long-term follow-up studies.

Data obtained is presented in Table 1. During the studied time-period, the relapse predictor was introduced. Thus, training, validation, and testing were performed by estimating the risk of relapse retrospectively. The annotation of the digital biomarker EE (2) during 2 or more consecutive days was used as ground truth and hence defines the concept “relapse”. An EE is defined as a rapid decrease in sobriety related indicator values (i.e., a rapid change to the worse) for a patient which in turn has been shown to correlate with physical biomarkers of alcohol consumption (2). Data collected before and after the introduction of the relapse predictor may differ. In retrospective analysis of data collected prior to the introduction of relapse prediction, caregivers and patients were of course not notified that relapse risk was increased. After the introduction relapse predictions were communicated to caregivers and patients allowing them to act and potentially prevent the predicted relapse. Messaging to a patient can occur in two ways. The caregiver can send a message at any point in time, and the eHealth tool can be configured to send an automatic message upon the relapse predictor determines that the risk for relapse is elevated. In total, data comprised 269,913 patient days from 1,809 patients before and 106,018 patient days from 933 patients after the introduction of the relapse predictor were analyzed (Table 1). The evaluated properties were relapse frequency (RPPD; Relapses per patient day), caregiver-patient messaging frequency, and relapse predictions. Their relationship to time and to each other were analyzed using regular descriptive statistics.

**Table 1:** Description of the data set.

Year	*N* patient days	*N* relapse days	*N* relapses	*N* predicted relapses	*N* caregiver messages
2015	372	158	39	–	–
2016	8,477	2,411	542	–	–
2017	31,805	7,411	1,825	–	–
2018	48,317	12,279	2,939	–	–
2019	63,334	13,778	3,447	–	–
2020	81,204	18,072	4,207	–	–
2021	83,696	20,608	4,679	1,354	2,553
2022	59,464	15,158	3,568	1,292	3,078
Total	376,669	89,875	21,246	2,646	5,631

*N* denotes “number of”. A relapse day is defined as a day with an exacerbation event. One relapse is defined as a continuous stretch of relapse days. Predicted relapses and caregiver messages were not implemented until 2021.

When the relapse predictor is activated, i.e., indicates that there is an elevated risk for relapse in the coming 1–3 days, the patient and caregiver will receive relapse warnings 3 consecutive days. This means that many performance measures can be calculated for either predictor activation or relapse warning. In this report, we analyze relapse risks over time with respect to predictor activation. The sensitivity, i.e., the fraction of actual relapses identified by the predictor, is however reported for prediction warnings, because this reflects the actual effect in clinical practice.

## 3 Results

**Figure 1:**
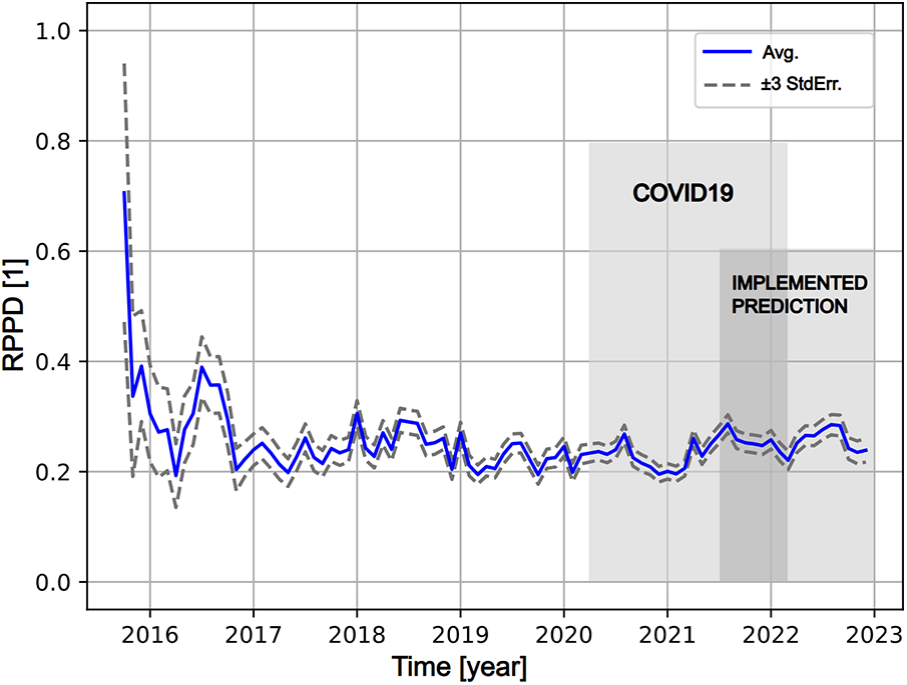
The average of relapse rate (RPPD; relapse per patient day) over time since introduction of previct alcohol (blue solid line) with estimated error range (±3*standard error, grey dashed lines). The start of the timeline represents Oct 1st 2015. The COVID-19 pandemic (March 2020–Jan 2022) is indicated as light grey. The predictor was implemented late in May 2021 and the associated period is indicated as light grey. The overlap between the pandemic and implementation is indicated as dark grey.

The relapse rate in the cohort of patients using the current eHealth tool is shown in Figure 1. After an initial high relapse rate of >0.4 RPPD in 2015 (3 months), the average relapse rate reached ≈ 0.25 RPPD in autumn 2016, decreased to 0.21 RPPD in 2020 and reached 0.25 RPPD in 2022. The COVID-19 restrictions 2020–2022 and the introduction of the relapse predictor May 2021 are indicated in Figure 1, and neither introduced any radical change on the average relapse rate.

**Figure 2:**
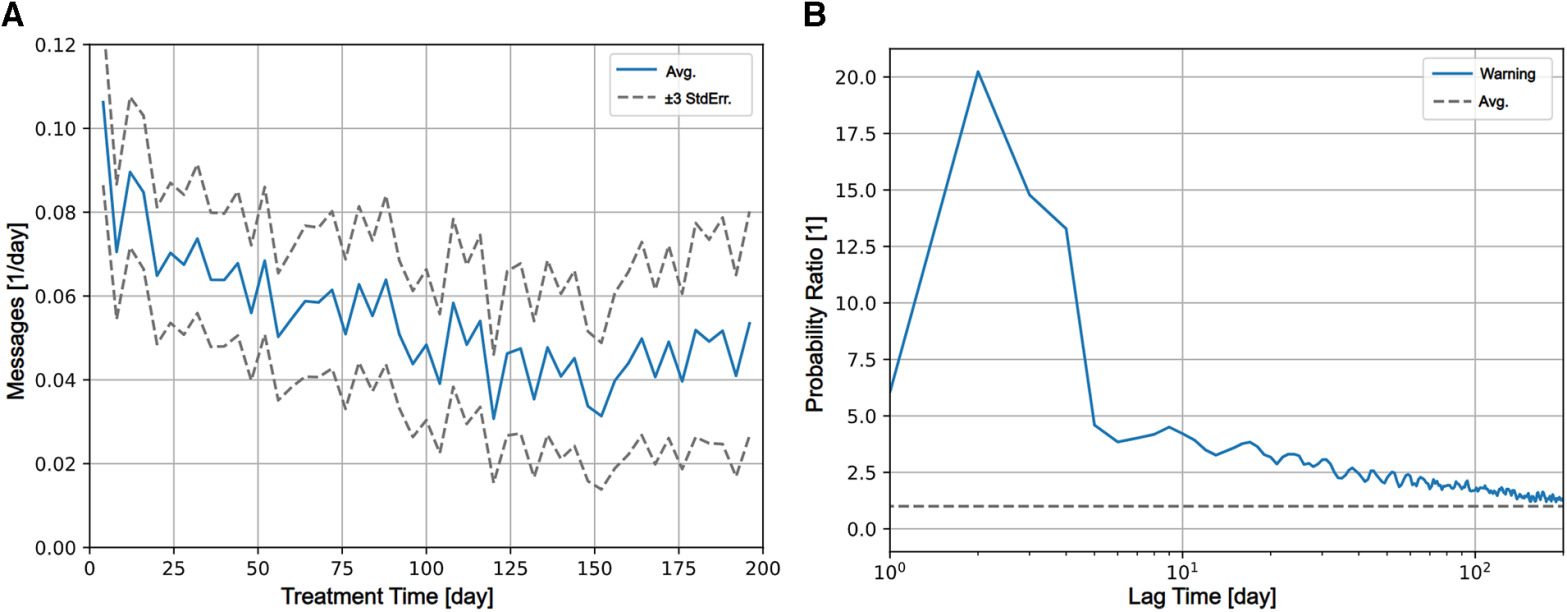
(**A**) Caregiver interactions through the eHealth tool (messages sent per day) decays over treatment time from an initial about 0.1 message sent per patient day to about 0.04 messages per patient day after 200 days in treatment. (**B**) The relative temporal patterns of coinciding events during treatment using cross-correlation are shown. Relative probability of messages sent to the patient (y-axis) at a lag of time (x-axis) after activation of the predictor (at day 0) is shown as blue solid line. The average probability for (any) message per patient day for the period was used for normalization is shown as dashed grey line. Days 1–3 after activation of the predictor, it is 10 times more probable that a message is sent, followed by a decline of messaging probability towards the average level.

**Figure 3:**
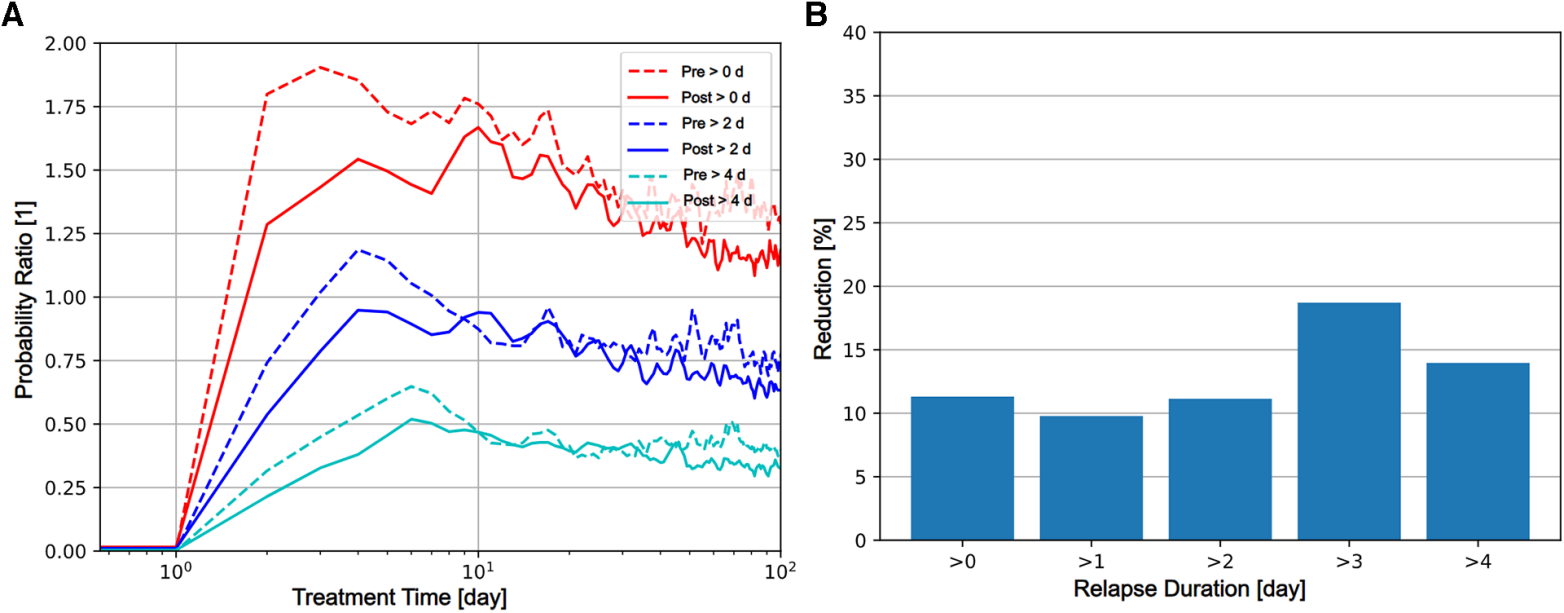
Coinciding events during treatment. The relative temporal patterns as investigated using cross-correlation are shown. (**A**) Relative risk (y-axis) of relapses of durations greater than 0, 2, 4 days observed for patients at a lag of time (x-axis) after activation of the predictor (at day 0) of are shown. Dashed lines indicate values computed from data collected from the “pre” period, prior to implementation of the predictor. Solid lines indicate values computed from data collected from the “post” period, after the implementation of the predictor. Red, Blue, and Cyan lines represent relapses greater than 0, 2, and 4 contiguous days. In the implementation of the predictor, activation cannot occur during a day in relapse. During the first 9 days following predictor activation, the probability of relapse is consistently smaller for “post” period data, indicating that the implementation of the predictor reduced the relapse quantity. (**B**) The relative reduction of risk for relapse in percent comparing the pre and post periods for duration (y-axis) greater than 0, 1, 2, 3, 4 contiguous days in relapse (x-axis) are shown as bars.

Messages to patients sent through the eHealth tool could be recorded only from May 2021 and onward, because the messaging feature was not implemented in early product versions. Messaging to patients is higher during the initial portion of the treatment, and gradually decays to about 1 message per 30 patient days after 200 days of treatment (Figure 2A). Messaging activity was unusually high 0–2 days after the notification was issued, mainly driven by automatic messaging (Figure 2B). The relapse rate increased sharply to clearly higher than usual after ∼3 days for the data before implementing the predictor, and ∼4 days (Figure 3A) with the prediction tool in production. The duration of relapses that occurred after a relapse warning were in general shorter after implementing the predictor (Figures 3A,B). The average RPPD shortly after an issued relapse warning was approximately 0.4 (pre) and 0.3 (post), meaning that more than every third warning resulted in a measurable relapse. Of all relapses, at least 1/6 were detected by the relapse predictor (seen as prediction warnings).

## 4 Discussion

The eHealth tool used under the present study—Previct Alcohol—has been depositing anonymous data to a data warehouse for the past 6 years. This database provides unprecedented possibilities for insights in the specific case of treatment support in AUD, but also represents eHealth tools in general.

A clear trait in all views of data is that the patient can, when enrolled in the eHealth tool under study, maintain relapse frequency at a constant level over time. The first year of operation presents highly fluctuating results (Figure 1) indicating that a new tool requires training and time to become functional, but after that the relapse frequency stabilizes. The relapse frequency from 2017 and onwards is an achievement in view of relapse frequencies reported by others (9, 11). It also corroborates that consistency and endurance in treating chronic conditions like AUD is of essence to keep adverse events low (13, 20). In a complicated disease like AUD where relapse may be considered the normal state, long term users of the eHealth tool evidently manage everyday life and maintain relapse frequency under control.

The individualization of care can be clearly illustrated and derived from the data. Upon the relapse predictor issuing a warning, there is an immediate effect seen in the communication with the patients. The database only registers the communication in the eHealth tools, and most probably the caregivers use other means of communication in parallel, e.g., calling or even visiting the patient at risk. The caregiver activity is provided timely, because shortly after the warnings were issued there is an actual increase in relapse rate (Figure 2), demonstrating that warnings are indeed related to increased risk for relapse. Even though only about 1/6 of all relapses are such that a relapse warning is issued, those cases that are warned for receive adequate attention by caregivers. The effect of issuing a warning for relapse is clear because the relapse quantity (in terms of onset and duration) is 9%–18% (Figure 3B) lower after the introduction of the relapse predictor. The issued relapse warning acts as help to recognize the early stages of relapse, a stage in which others (14) claim that the chances of successful prevention are greatest. Our findings support this view and quantify the effect in terms of reduction of relapse quantity and duration.

The ability to measure is an important point of eHealth tools. Population level relapse frequencies can be depicted over time, relapse patterns can be evaluated, geographic regions can be compared and so on. Even though the quality of data is high, and the quantity of data is massive, there are two major challenges in evaluating the eHealth system in this study. Firstly, what can the observations be compared to? The lack of objective and systematic measurement outside the Previct Alcohol user group means that only rough estimates are available as comparator. The average RPPD for Previct Alcohol is 0.22, which we believe is clearly lower than any other similar cohort with a traditional treatment program. Lack of measurement on traditional cohorts means that we cannot be certain. Secondly, how can one assess the efficiency in the alleviation of an event, in our case prevention of relapse? Among the 1,354 warnings issued in 2021, 384 were followed by a confirmed relapse (Table 1). Since the caregivers and patients were notified, they had the opportunity to intervene. If the predictor had not been implemented, we estimate that this number would be in the order of 420–460 confirmed relapses. The savings in terms of number of days in relapse cannot be estimated at this point in time.

Any prediction tool should be tuned adequately to aid when adequate without overwhelming the operator. When placed in its operating environment, the relapse prediction would provide the following aid and burden in a typical case: The average RPPD is approximately 1/5 and the number of relapse days is 4–5 times larger than the number of relapses (Table 1). For an average patient this would mean one 5-day relapse each month. About every third prediction represents a measurable relapse which means that for each accurate prediction, another two prediction warnings will be issued. With a patient enrolled 6 months, and a sensitivity of ∼1/6, one relapse would be accurately predicted and potentially alleviated, and another two prediction warnings would be issued. The eHealth system is conservatively tuned and the risk of overwhelming the caregiver is low. Also, the predictor performance combined with its the conservative tuning means that the number of alleviated relapses is small in comparison to the total number of relapses. Hence, the effect of relapse prediction in view of average relapse rate (such as in Figure 1) would be comparable to the fluctuations of RPPD between 2017 and 2023. It could be advisable to revisit the thresholds of the predictor and allow an increased number of prediction warnings to bring greater effect while adding some burden to the caregiver.

From a clinical standpoint, use and abuse of alcohol is a large public health problem world-wide. Understanding where and when to interact to avoid the damage and suffering from a relapse is of vital importance for the health care provider. Whereas treating all patients within the healthcare system to avoid a relapse is not possible, an eHealth tool like Previct Alcohol will allow more precise use of resources where they are needed. This is clearly seen in the fact that caregiver activity in average decreases over time except when relapse warnings are issued (Figure 2), but the average relapse rate stays the same in the cohort (Figure 1). Considering that active contact with the patient is important in the management of AUD, even a technically inaccurate a prediction warning serves as a reminder to reach out and check the status of the patient, hence potentially contributing to clinical effect. Altogether, prediction warnings will improve patients health and reduce the suffering of relatives, while at the same time spending less resources.

From the patient perspective, fewer and/or shorter relapses are important to save the patient's social and economic situation: With fewer days of sickness leave and rehabilitation after a relapse, the risk of losing employment is reduced. An eHealth tool can also engage family members and provide digital support at any time in a patient-centric manner. Further, patient engagement should not be underestimated, where a strive to stay compliant with scheduled alcohol tests may become a health-promoting goal in itself. In this aspect, adequately designed eHealth tools empower the patient to manage their own disease to achieve a higher level of social function. Even though this study is based on anonymous and retrospective data, the results seen would not have been possible unless the tool has sufficient usability to be mastered by the patient, and sufficient efficacy in its mode of action. eHealth tools like Previct Alcohol can be a gamechanger for patients with AUD.

In sharp contrast to reports by others (18, 19), COVID-19 did not affect the monitored patient population in any dramatic manner (Figure 1). This observation could imply that the eHealth tool served as a sufficiently protective measure for the patients also under the unusual circumstances of COVID-19. The eHealth tool was used in a distributed manner long before COVID-19, which means that both the enrolled patients and the caregivers were already used to remote care. Under all circumstances, the patients enrolled in the eHealth tool during COVID-19 times managed their disease in a good manner.

This study has several strengths and limitations. The main strength is that the data warehouse contains a large data set with real-world data collected in clinical practice with consistent data collection over a period of many years. The data warehouse is, however, due to the requirement of being anonymous, limited in some respects. It reflects live use of the eHealth tool, good in the aspect of a real-world source of data but challenging because it is not designed to test a hypothesis. It is an isolated data set, with information available in the patient journals and similarly being inaccessible, meaning there is no record of what actions the caregivers took in response to output from the eHealth tool. The cohort under study is predominantly from Sweden. Data was collected before, during and after COVID-19, and the introduction of the prediction tool overlapped with COVID-19. Further, AUD is a disease known to have large individual variation. With these shortcomings in mind, all statistical claims underwent a two-stage analysis on different subsets of data to avoid overfitting. This strategy alleviates some, but not all, weaknesses in data. Known limitations are continuously addressed, where possible, in that the data transfer process to the data-warehouse is updated regularly, but any updates will only apply to data transferred after each update.

It would be possible to design clinical studies to allow more precise statements on the performance of the eHealth tool, which would resolve the issues related to the real-world character of data. The field of AUD treatment would benefit from investigation of basic characteristics, like the average RPPD for conventional treatment programs, this to estimate the effect of advanced tools like the one discussed in the present report in proper light. The design of such a clinical study would probably encompass a multi-center randomized controlled clinical study, including before-after analysis. The overall effect characteristics of each study center could be characterized before and after the start of the study, and two arms (treatment as usual and implementation of an advanced therapy support tool) could be compared. Another important topic would be to focus on relapse prediction and relapse alleviation, where data is collected to refine the AI driven prediction tool as well as suitable caregiver procedures following a relapse prediction are studied to provide insight into what most efficiently breaks the patient trajectory towards a predicted relapse. All suppliers of medical devices to the European Union are obliged to regularly follow up the performance of their products. Sometimes, the follow-up results in valuable insights that can be shared in scientific literature, such as the findings reported here. Regular follow-up of the eHealth tool under study will be conducted in the future. We foresee more precise yet anonymous data collection, with focus describing the relationship between care activities and benefits for the patient. For example, the impact of self-reported perceived well-being and motivation on the recovery process could be analyzed. The life-cycle of a medical device can be seen as an iterative learning activity where use is perfected by continuous observation and change.

In conclusion, the ability to follow a disease through the eyes of an eHealth tool brings large-scale, data rich, population wide findings into light. In our case, predicted relapses have been clearly reduced and therapists receive support to provide precision treatment through a relapse prediction tool. Introduction of the eHealth tool for supporting the treatment of AUD brings value both in the ability to measure effects and in generating effects. With the prediction warnings proven adequate and efficacious, care programs where caregivers follow pre-defined action plans upon receiving a prediction warning should be evaluated to increase efficacy further. Consistency and endurance are key for implementing eHealth systems like the one under study and the effects can be clear: During 2021–2022, 2,646 relapse warnings were issued, allowing precise caregiver action which kept relapse rates in control and reduced relapse quantity, this to the clear benefit of patients, family, and society. We speculate that the practical effect of the reduction in relapse translates to more children having sober parents, fewer spouses being subjected to in-relationship violence, less sickness-leave, and fewer in-patient rehabilitation sessions paid by health care funds.

## Data Availability

The original contributions presented in the study are included in the article/Supplementary Material, further inquiries can be directed to the corresponding author.
